# SPOUSAL CAREGIVING AND PARALLEL COGNITIVE TRAJECTORIES AMONG OLDER ADULT CAREGIVERS AND RECIPIENTS IN CHINA

**DOI:** 10.1093/geroni/igad104.2646

**Published:** 2023-12-21

**Authors:** Guo Yu, Zhenmei Zhang, Quanbao Jiang

**Affiliations:** Xi’an Jiaotong University, Lansing, Michigan, United States; Michigan State University, East Lansing, Michigan, United States; Xi’an jiaotong university, Xi‘an, Shaanxi, China (People’s Republic)

## Abstract

Prior studies have paid much attention to the physical and mental health outcomes of spouse caregivers. However, few have looked at the cognitive trajectory of caregivers and how it was associated with the characteristics of caregivers and care recipients. Based on four waves (2011, 2013, 2015, and 2018) of the Chinese Health and Retirement Longitudinal Survey (CHARLS) (N=3137 dyad couples aged 45 and above), cognitive function was measured by Mini-Mental State Examination (MMSE). A parallel process growth model was conducted to explore the association between spouse caregiving and the joint trajectories of caregivers and their recipients’ cognitive decline during the eight years. Preliminary results show that 1) couples’ trajectories are significantly related to each other, although men had a higher initial level of cognitive function than women, their cognitive function declined at a faster rate than women did; 2) The relationship between caregiving and cognitive trajectory varies by gender. Specifically, providing care is positively correlated with slower cognitive decline in men, but not in women. Whereas receiving the care was associated with a faster cognitive decline for both gender. These findings emphasize the need of paying attention to both family caregivers and recipients and the need for more targeted intervention strategies based on the nature of care.

